# Targeting Matrix Metalloproteinase-3 for Dental Caries Prevention Using Herbal Isolates: MMP3 Inhibition by Cinnamic Acids

**DOI:** 10.1155/2024/9970824

**Published:** 2024-10-08

**Authors:** Mahdieh Salman, Bahareh Asgartooran, Amir Taherkhani

**Affiliations:** ^1^Department of Restorative Dentistry, School of Dentistry, Hamadan University of Medical Sciences, Hamadan, Iran; ^2^Research Center for Molecular Medicine, Hamadan University of Medical Sciences, Hamadan, Iran

**Keywords:** cinnamic acid, docking, drug, inhibitor, matrix metalloproteinase-3

## Abstract

**Objectives:** Dental caries, a prevalent infectious disease affecting teeth, ranks highest among 328 diseases, according to a 2017 Lancet study. In demineralized human dentin, matrix metalloproteinase-3 (MMP3) functions as a proteoglycanase, contributing to the degradation of proteoglycan components. This process exposes collagen fibrils, thereby facilitating the demineralization of the dentin matrix. Inhibiting MMP3 shows potential for preventing dental caries.

**Methods:** The binding affinity of 20 cinnamic acid derivatives, namely cynarin, chlorogenic acid, rosmarinic acid, cinnamyl caffeate, phenethyl caffeate, N-p-coumaroyltyramine, caffeic acid 3-glucoside, caffeic acid phenethyl ester, roscovitine, benzyl caffeate, o-coumaric acid, artepillin C, caffeic acid, methyl caffeate, 2-methylcinnamic acid, ferulic acid, drupanin, p-coumaric acid, cinnamic acid, and sinapinic acid, to the MMP3 catalytic cleft, was assessed utilizing AutoDock 4.0. Molecular dynamics simulation was then employed to analyze the stability of backbone atoms in free MMP3, MMP3-positive control inhibitor, and MMP3 complexed with the top-ranked cinnamic acid over a 100 ns computer simulation.

**Results:** Four cinnamic acids demonstrated *ΔG*_binding_ scores below −10 kcal/mol, with cynarin emerging as the most potent MMP3 inhibitor, featuring a *ΔG*_binding_ score and inhibition constant value of −15.57 kcal/mol and 3.83 pM, respectively. The MMP3–cynarin complex exhibited stability after a 50 ns computer simulation, showing a root-mean-square deviation of 8 Å.

**Conclusions:** The inhibition of MMP3 by cynarin, chlorogenic acid, rosmarinic acid, and cinnamyl caffeate holds promise as a potential preventive strategy for dental caries.

## 1. Introduction

Dental caries, the most prevalent infectious disease affecting dental hard tissues, ranks the highest among 328 diseases [1]. Key factors include bacteria, carbohydrates, saliva composition, and poor hygiene [2–4]. The ecological plaque hypothesis explains caries formation through an oral microflora imbalance, promoting *Streptococcus mutans* and Lactobacillus overgrowth [5, 6]. Elevated lactic acid levels demineralize enamel and activate matrix metalloproteinases (MMPs), leading to cavity formation. The bacterial biofilm progresses through attachment, irreversible attachment, and matrix production, forming a three-dimensional structure [7].

Proteolytic activities, conventionally attributed to bacterial proteases, encompass activated endogenous MMPs found in dentin, gingival crevicular fluid, and saliva. MMPs are a group of over 25 zinc-containing endopeptidases that play a pivotal role in the degradation of demineralized dentin matrix at neutralized pH levels, extracellular matrix dynamics, tooth formation, degradation, and periodontal diseases [8–12]. In active carious tooth sites, bacterial collagenases and endogenous MMPs contribute to the destruction of the dentin matrix. Local pH changes activate dormant MMPs with diverse origins, including salivary, gingival fluid, and dentinal sources. Although the precise role of MMPs from different origins remains unclear, studies suggest the involvement of dentinal fluid as a source of MMPs and cathepsins [13, 14]. Implicated in dental caries and restoration failure, MMPs include MMP1, MMP2, matrix metalloproteinase-3 (MMP3), MMP8, MMP9, and MMP20 [13]. MMP3, identified in demineralized human dentin, functions as a proteoglycanase, inducing proteoglycan destruction. Its proteolytic activity occurs specifically in demineralized dentin, and studies have also detected MMP3 in the predentine layer and dentinal tubules of bovine molars [15–17]. As a stromelysin, MMP3 does not target collagen but cleaves proteoglycans and glycoproteins. Two main types of proteoglycans are present in dental tissue—one associated with predentine tissue, acting as a mineralization inhibitor, and the other at the mineralization front within the dentin, serving as a mineralization promoter [18, 19].

Cinnamic acid is an aromatic carboxylic acid found abundantly in nature, derived from cinnamon, ginseng, certain fruits, whole grains, vegetables, and honey [20, 21]. Recent research demonstrates that cinnamic acid possesses promising antioxidant, antimicrobial, anticancer, neuroprotective, anti-inflammatory, and antidiabetic activities [22, 23]. These varied bioactivities likely arise from the carboxyl and aromatic functional groups characteristic of cinnamic acid, enabling intermolecular interactions within complex biological systems [22–25]. Further investigation of this naturally occurring small molecule may provide novel therapeutic applications in multiple disease states [20, 21]. By elucidating the mechanisms underlying cinnamic acid's multitargeted biological effects, continued studies in this area can enable translation from bench to bedside [22, 23].

Prior research has established a strong binding affinity between several cinnamic acids, MMP8 [26] and MMP9 [23]. Our current study postulates that these herbal isolates may also serve as potential inhibitors of MMP3. Our investigation focused on examining the inhibitory impact of selected cinnamic acids on the MMP3 catalytic cleft. Utilizing AutoDock 4.0, we assessed the binding affinity of this class of herbal compounds to the MMP3 catalytic cleft. Additionally, molecular dynamics (MDs) simulations were conducted to evaluate the stability of MMP3 when bound to our top-ranked cinnamic acid, compared to free MMP3 and MMP3 with a positive control inhibitor, referred to as marimastat.

## 2. Materials and Methods

### 2.1. Structural Preparation

To obtain a high-resolution structure of MMP3 for this study, the publicly accessible Protein Data Bank from the Research Collaboratory for Structural Bioinformatics (RCSBs) [27] was utilized. Applying a filter for X-ray crystallographic data at 1.5-Å resolution or better, the MMP3 structure with PDB identifier 1HY7 [28] was selected and retrieved, determined initially at 1.5 Å resolution using X-ray diffraction methods. By leveraging this specific three-dimensional structural data from RCSB, further computational analyses could be undertaken to better understand the structural basis of the MMP3 function. Access to such intricate molecular data in the PDB supports advanced biophysical techniques and Impact modeling of therapeutic targets like MMP3.

Subsequently, the obtained structures were visualized utilizing BIOVIA Discovery Studio Visualizer (DSV). The acquired MMP3 protein structure contained two polypeptides labeled A and B per PDB conventions. Chain A comprised 167 residues, spanning from residue 83 to 250. In molecular docking simulations, emphasis was placed on chain B, aligning with the structural representation of 1HY7 and encompassing 172 amino acids from residue 583 to 755. The processing of the PDB file involved utilizing Notepad++ to systematically eliminate nonprotein atoms, such as the control inhibitor (PDB ID: MBS; PubChem ID: 446165; Drugbank ID: DB04416). The analysis of the MBS docked pose was conducted using the DSV tool, facilitating a comprehensive examination of the interaction from a two-dimensional perspective. This approach enabled the discernment of residues within the active site of 1HY7 that engaged with MBS. To attain the almost stable 1HY7 structure, 100 energy minimization steps were executed using Swiss-PdbViewer, available at https://spdbv.unil.ch [29]. Herein, a comprehensive analysis was conducted on a set of 20 well-established cinnamic acids, encompassing cynarin, chlorogenic acid, rosmarinic acid, cinnamyl caffeate, phenethyl caffeate, N-p-coumaroyltyramine, caffeic acid 3-glucoside, caffeic acid phenethyl ester, roscovitine, benzyl caffeate, o-coumaric acid, artepillin C, caffeic acid, methyl caffeate, 2-methylcinnamic acid, ferulic acid, drupanin, p-coumaric acid, cinnamic acid, and sinapinic acid [30]. These compounds had previously undergone evaluation against MMP8 [26] and MMP9 [23]. The affinity of the identified drug candidate to MMP3 was systematically compared with that of MBS, along with marimastat serving as the positive control inhibitor for MMP3 inhibition. Additionally, the HyperChem software version 8.0.10 [31] was used to attain the most stable formation of the small molecules under investigation.

### 2.2. Molecular Docking Analysis

The docking analyses were conducted using a computer with a Windows operating system. The computer had an Intel Core i7 central processing unit, 16 gigabytes of random access memory, and a 64-bit system architecture [32]. The *ΔG*_binding_ values of the interactions between cinnamic acids, positive control inhibitors, and the MMP3 catalytic cleft were calculated using AutoDock 4.0 and a semiflexible docking technique [33]. The two-dimensional representation of the interactions between MMP3 and MBS revealed 18 residues within the enzyme's active site. These residues include Tyr655, Val663, Leu664, Ala665, His666, Ala667, Leu697, Val698, His701, Leu702, His705, His711, Ala717, Leu718, Tyr720, Pro721, Leu722, and Tyr723. Accordingly, the grid box options were configured with the following parameters: *X*-dimension at 52, *Y*-dimension at 48, *Z*-dimension at 74, *X*-center at 0.231 Å, *Y*-center at 49.256 Å, and *Z*-center at 53.451 Å. Fifty docked poses were systematically generated for each compound utilizing the Lamarckian Genetic Algorithm to evaluate the binding affinity among the investigated compounds and the MMP3 active site. The *ΔG*_binding_ values derived from these models were subjected to clustering with a root-mean-square (RMS) tolerance set at 2 Å. The most negative *ΔG*_binding_ score inside the predominant cluster was subsequently chosen for comprehensive assessment. Following this, the DSV tool was employed to construct molecular images.

### 2.3. MD Simulations

The MD simulations, spanning a duration of 100 nanoseconds (ns), were executed utilizing the Discovery Studio Client tool. The primary goal was to assess the structural stability of the most salient complex in comparison to the positive control inhibitor [34–36]. The simulations were performed using a high-powered computing system with specifications including a 64-bit operational framework, 64 gigabytes of DDR5 random access memory, and a 24-core Intel i9-13900KF central processing unit, representing a computational configuration that surpassed that utilized in the earlier docking analyses.

The configuration of the simulations incorporated defined parameters, including a 310 K target temperature, the CHARMm force field, water as the solvation solvent, an orthorhombic unit cell geometry, a 10 Å minimum separation distance from the cell boundary, an explicit periodic boundary condition for the solvation method, and usage of a point charge allocation. The results obtained from MD simulations for the preeminent complex were systematically contrasted with those involving the free MMP3 and the enzyme inhibited by the positive control inhibitor.

### 2.4. Interaction Mode Analysis

The most promising MMP3 inhibitors were selected by assessing their *ΔG*_binding_ values. Compounds exhibiting binding energy below −10 kilocalories per mole (kcal/mol) were identified as the most potent MMP3 inhibitors. These chosen compounds subsequently underwent interaction mode evaluation utilizing the DSV instrument, and the results were compared to those of the control inhibitors. For the highest affinity MMP3 inhibitor, the observed interactions were compared to those distinguished after performing MD simulations.

## 3. Results

### 3.1. Binding Affinity Assessment

**Figure 1:**
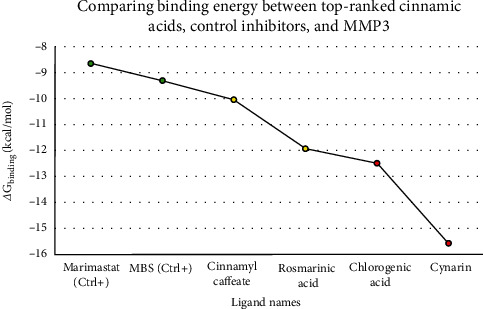
The Gibbs free binding energy, as denoted by the Δ*G*_binding_, values measured in kcal/mol units, delineates the interactions among the foremost cinnamic acids, positive control inhibitors for 1HY7, and the receptor's catalytic cleft. Compound names are depicted on the *X*-axis, while the corresponding Gibbs free binding energy is represented on the *Y*-axis in the graphical presentation. The red and yellow regions are proposed to exert inhibitory effects on MMP3 activity at the picomolar and nanomolar scales. Concurrently, the green spheres represent inhibitors serving as positive controls.

By assessing the *ΔG*_binding_ parameter for the catalytic cleft in the presence of four cinnamic acids, registering a value below −10 kcal/mol, the foremost inhibitors for the 1HY7 target, as ascertained in this investigation, were discerned. The efficacy of cynarin, chlorogenic acid, rosmarinic acid, and cinnamyl caffeate as potent inhibitors for 1HY7 was underscored by their respective calculated binding energy values: −15.57, −12.48, −11.93, and −10.04 kcal/mol. In light of the estimated inhibition constant (*K*i) values, it was observed that cynarin and chlorogenic acid manifested a remarkably potent inhibitory impact on MMP3, registering values within the picomolar (pM) range. Furthermore, the inhibition constant values attributed to rosmarinic acid and cinnamyl caffeate were at the nanomolar (nM) concentration. The computation of the Δ*G*_binding_ score involving marimastat, MBS, and MMP3 revealed that the preeminent cinnamic acids in this investigation exhibited heightened binding affinities to the 1HY7 active site compared to the control inhibitors. Specifically, the *ΔG*_binding_ values between marimastat, MBS, and MMP3 were determined as −8.65 and −9.3 kcal/mol (Figure 1).  Table 1 delineates the computed *ΔG*_binding_ and *K*i values in the interactions between 1HY7 and the entire array of tested ligands. A comprehensive scrutiny of the top-ranking cinnamic acids and the 1HY7 catalytic cleft was conducted, with a detailed presentation of all computed energy types provided in Table 2.

### 3.2. MD Simulations

**Figure 2:**
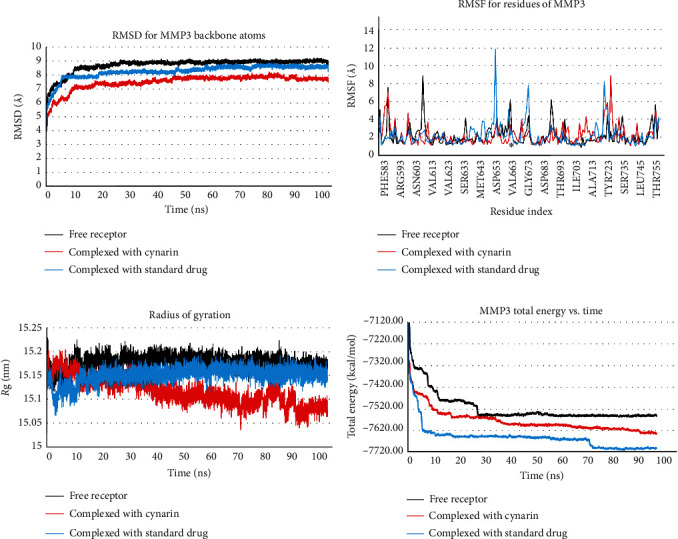
The impact of cynarin and marimastat on MMP3 backbone atoms was thoroughly examined through an exhaustive 100 ns molecular dynamics simulation, with specific attention to (a) RMSD, (b) RMSF, (c) ROG, and (d) total energy plots. Notably, asterisks within the RMSF plots indicate the locations of the active site on the receptor. MMP3, matrix metalloproteinase-3; RMSF, root-mean-square fluctuation; RMSDs, root-mean-square deviations.

The MD simulations have yielded valuable insights into the dynamic behavior of MMP3 in the presence of cynarin and marimastat. Notably, the RMS deviations plot (refer to Figure 2a) has unveiled a significantly more stable MMP3 structure when complexed with cynarin, compared to both the MMP3–marimastat complex and the free receptor. The backbone atoms of MMP3 achieved a stable conformation approximately 50 ns into the simulation, maintaining a consistent level of approximately 8 Å in the presence of cynarin. Furthermore, the complex with marimastat exhibited stability after 60 ns, with the protein's backbone atoms stabilizing at approximately 8.5 Å.

An examination of the RMS fluctuation plot (see Figure 2b) highlighted enhanced stability of MMP3′s backbone atoms, particularly within the enzyme's active site (Val663), when complexed with cynarin, in contrast to the MMP3–marimastat complex and the free enzyme. Subsequent in-depth MD analyses disclosed that following a 40 ns simulation, the MMP3 radius of gyration (ROG) was significantly lower in the presence of cynarin compared to marimastat and the free enzyme, as depicted in Figure 2c. Additionally, the MMP3 total energy consistently remained lower throughout the entire simulation period when in complex with marimastat, followed by MMP3–cynarin and the free receptor, as illustrated in Figure 2d.

### 3.3. Interaction Modes

**Figure 3:**
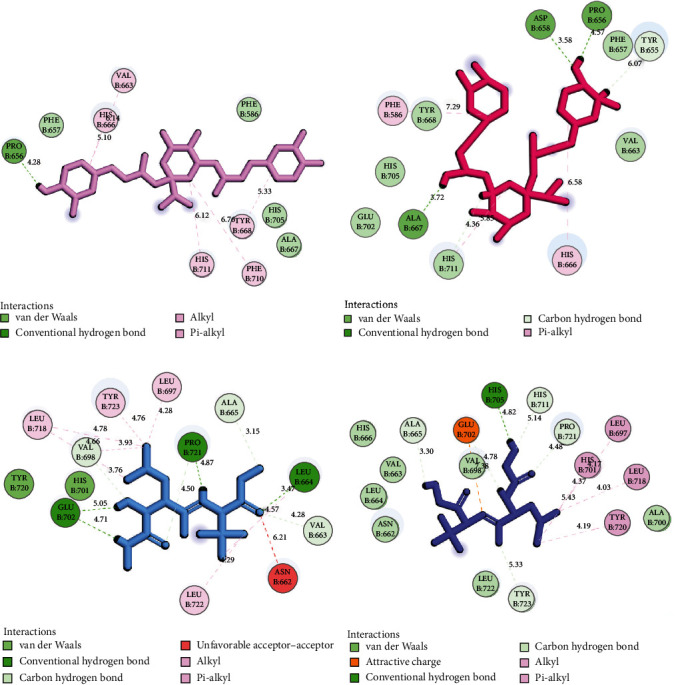
Two-dimensional view of cynarin within the MMP3 active site before (a) and after (b) MD simulations. The incorporation of marimastat inside the MMP3 catalytic domain is shown before (c) and after (d) MD simulations. MMP3, matrix metalloproteinase-3; MDs, molecular dynamics.

**Figure 4:**
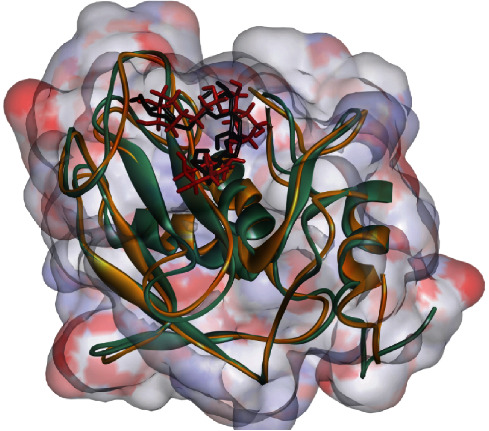
The superimposed structure of MMP3 in the presence of cynarin throughout a 100 ns MD simulation is illustrated. The protein is depicted in green and brown chains, representing its state before and after MD analysis. Additionally, the ligands are shown in black and red colors, signifying their configurations before and after MD simulations, respectively. MMP3, matrix metalloproteinase-3; MDs, molecular dynamics.

Within the scope of ligands investigated, rosmarinic acid manifested the formation of the highest number of H-bonds (*n* = 5). At the same time, cinnamyl caffeate exhibited the most extensive hydrophobic connections (*n* = 9) with the 1HY7 residues. Preceding the MD simulation, cynarin, identified as the most potent inhibitor of 1HY7, established contact with the active site through one hydrogen bond and four hydrophobic interactions. After the MD simulation, this metabolite displayed an augmented interaction profile, engaging in four H-bonds and three hydrophobic interactions with the MMP3 residues. A comprehensive account of the interactions between the ligands and the residues constituting the 1HY7 active site is presented in Table 3. Additionally, Figure 3 furnishes a two-dimensional visualization delineating the interactions between cynarin, marimastat, and the MMP3 residues. At the same time, Figure 4 provides a three-dimensional depiction illustrating the blockade of MMP3 by cynarin, both before and after MD simulations.

## 4. Discussion

The critical role of proteoglycans in the dentin matrix is warranted, as highlighted in the study by CdMP et al. [37]. Proteoglycans are shown to exert pivotal regulatory functions in dentin biomineralization, offering mechanical support and compressive strength to the mineralized tissue. Through enzymatic digestion protocols, CdMP et al. [37] demonstrated that the selective removal of glycosaminoglycan chains and proteoglycans from demineralized dentin significantly influences the bulk mechanical properties and biostability of the type I collagen matrix. Specifically, trypsin-mediated proteoglycan digestion reduced dentin tensile strength, while chondroitinase ABC treatment decreased the energy required for fracture. These findings underscore proteoglycans' indispensability in preserving the dentin matrix's structural integrity and biomechanical attributes. In our study, inhibiting MMP3, a proteoglycanase in demineralized dentin, may aid in safeguarding the proteoglycan-mediated stabilization of the collagen network, potentially enhancing remineralization capacity and overall resistance against carious lesion formation.

Traditional drug discovery is time-consuming and expensive [38]. Computer-aided methods, particularly structure-based and ligand-based drug design, offer cost-effective alternatives. Molecular docking, a structure-based approach, utilizes biological receptor data, while ligand-based design relies on existing knowledge of ligand interactions [39]. Dental caries is a multifactorial process that can lead to tooth loss and burdens on individuals and society. A wide variety of herbal products demonstrate anticariogenic activity through various procedures. Recent studies illustrate the anticariogenic effect of cinnamic acid derivatives by inhibiting MMPs, including MMP8 and MMP9 [23, 26, 32]. This study employed a structure-based in silico method to identify innovative MMP3 inhibitors from Cinnamic acids.

The findings of the current investigation unveiled that cynarin, chlorogenic acid, rosmarinic acid, and cinnamyl caffeate demonstrated significant binding affinity with the active site of MMP3. Cynarin, an artichoke-derived phytochemical, exhibits various pharmacological attributes, encompassing free-radical scavenging, antimicrobial, and antihistaminic activities [40, 41]. Cynarin possesses biologically active functional groups derived from hydroxycinnamic acid [42]. Within the scope of the current research, cynarin exhibited the highest binding affinity to MMP3 compared to the other cinnamic acid derivatives discussed. This pronounced affinity was evidenced by a binding energy value of −15.57 kcal/mol. Cynarin established hydrogen bonds with the Pro656 amino acid and engaged in hydrophobic interactions with Val663, His666, His711, and Tyr668. Elmosallamy et al. [43] illustrated that artichokes harbor a spectrum of phytochemicals, encompassing polyphenols such as cynarin, caffeoylquinic acid, chlorogenic acid, and several flavonoids, including luteolin and glycosides. Their investigation unveiled that the administration of artichoke resulted in a notable reduction in the elevation of liver enzymes and oxidative stress. Furthermore, the treatment induced apoptosis by inhibiting MMP3, MMP9, and MMP12 [43]. This observation aligns with the results obtained in our study.

The current study unveiled that chlorogenic acid yielded the second-highest binding affinity toward the active site of the MMP3 molecule, demonstrating a binding energy value of −12.48 kcal/mol. This interaction was characterized by hydrogen bonds formed with Val663, Leu664, Pro721, and Asn662 amino acids and hydrophobic interactions involving Val698 and His701. This metabolite is one of the most abundant cinnamic acids within phenolic compounds, naturally occurring in green coffee extracts and tea. As a notable biologically active dietary polyphenol, it plays a multifaceted role in various therapeutic capacities, including antioxidant activity, anti-inflammatory actions, and antimicrobial activity [44]. Lee et al. [45] documented that intraperitoneal injection of chlorogenic acid exhibited radical scavenging activity and demonstrated inhibitory effects on MMP2 and MMP9, resulting in reduced infarct volume and alleviation of sensory–motor functional deficits. According to the experimental validation by Lee et al. [45], the potential efficacy of chlorogenic acid in inhibiting MMP3 might be suggested. However, future studies need to validate chlorogenic acid's specific inhibition of MMP3.

Rosmarinic acid is a natural ester derived from caffeine acid and 3,4-dihydroxy phenyl lactic and can be found in nature as a phenolic compound. It is mainly derived from species Boraginaceae family, subfamily Nepetoideae [46]. Recent studies have proven that rosmarinic acid in medicinal plants, herbs, and spices positively correlates with remarkable biological advantages [47]. In the present study, rosmarinic acid demonstrates the binding energy of −11.93 toward the active site of 1HY7, forming hydrogen bonds with Asn662, His711, and Tyr723 amino acids and hydrophobic interaction with His701, Val698, and Val663 residues. Chen et al. [48] conducted a study on rat chondrocytes, revealing that rosmarinic acid downregulated MMP1, MMP3, and MMP13 expression. In a separate investigation, Eo and Kim [49] focused on rabbit articular chondrocytes, observing that rosmarinic acid decreased the expression of MMP13 and suppressed the degradation of extracellular matrix components. Furthermore, Liu et al. [50] reported that rosmarinic acid effectively diminished the expression of MMP2 and MMP9 in human glioma cells.

Numerous studies have underscored the notable antimicrobial and biofilm inhibitory properties exhibited by specific cinnamic acids, particularly caffeic acid phenethyl ester, chlorogenic acid, and cinnamic acid, against prevalent dental caries pathogens like *S. mutans* and *Candida albicans* [51, 52]. Caffeic acid phenethyl ester's demonstrated efficacy in impeding cross-kingdom biofilm formation of *S. mutans* and *C. albicans*, along with its modulation of cariogenic genes, underscores its potential as a promising antimicrobial agent [51, 52]. Similarly, chlorogenic acid's capacity to stimulate osteogenic differentiation of human dental pulp stem cells via Wnt signaling [53] and its impact on microbial communities within dental unit waterlines [54] further support its significance in dental health maintenance and disease prevention. Additionally, the utilization of caffeic acid in orchestrating the interplay between antimicrobial and anti-inflammatory responses in macrophages against *S. mutans* infection [55] showcases the multifaceted benefits of these compounds beyond their antimicrobial attributes. A novel approach utilizing photoirradiated caffeic acid has demonstrated bactericidal effects against *S. mutans* biofilms through hydroxyl radical formation [56], suggesting a potential avenue for augmenting the antimicrobial efficacy of phenolic compounds through physical methods. Furthermore, the regenerative potential of caffeic acid, exemplified by its promotion of vascular endothelial growth factor production in human dental pulp cells [57], and the osteogenic and angiogenic enhancements offered by caffeic acid-inspired mineral trioxide aggregate scaffolds [58], underscore the promise of these compounds in dental tissue repair and regeneration. A previous report by Ribeiro et al. [59] explored the antimicrobial effect of cinnamic acid against *S. mutans*. Furthermore, functionalizing zinc oxide nanoparticles with cinnamic acid to target dental pathogens and modulate apoptotic genes in oral epidermal carcinoma cells [60] introduced an innovative nanotechnology-based strategy for managing dental pathogens and preventing oral cancer.

While this study has shown promising inhibitory effects of selected cinnamic acid derivatives against the MMP3 enzyme, it is important to acknowledge several limitations. First, this evaluation was conducted in silico, necessitating further studies to validate the efficacy of these compounds in more clinically relevant models, such as dental caries lesion formation assays or remineralization studies using extracted teeth. Additionally, a thorough investigation of top-performing inhibitors' bioavailability and pharmacokinetic properties, such as cynarin, is essential to assess their potential for real-world clinical applications. Furthermore, exploring the synergistic effects of combining MMP3 inhibition with other remineralization strategies could offer fruitful avenues for future research. Ultimately, the overarching goal is to translate these findings into innovative preventive or therapeutic interventions to address the global burden of dental caries.

## 5. Conclusion

The findings of this study indicate that cynarin, chlorogenic acid, rosmarinic acid, and cinnamyl caffeate exhibit substantial inhibitory effects on MMP3, with cynarin and chlorogenic acid emerging as the most salient metabolites, displaying a *K*i score at the pM concentration. Additionally, the MMP3 conformation remained stable throughout approximately 50 ns of computer simulation when bound with cynarin. These results hold promise for guiding scientists in developing potential drugs for combating dental caries or incorporating novel formulations into toothpaste and mouthwashes. Nevertheless, it is crucial to note that further validation through in vitro and in vivo experiments is imperative to corroborate these results.

## Figures and Tables

**Table 1:** The evaluation of Gibbs free energy and *K*i values for the active site of 1HY7 was conducted in the presence of 20 cinnamic acids, accompanied by two positive control inhibitors.

PubChem ID	Ligand name	Binding energy (kcal/mol)	*K*i
6124212	Cynarin	−15.57	3.83 pM
1794427	Chlorogenic acid	−12.48	707.54 pM
5281792	Rosmarinic acid	−11.93	1.79 nM
11380911	Cinnamyl caffeate	−10.04	43.95 nM
5281787	Phenethyl caffeate	−9.98	48.56 nM
5372945	N-p-Coumaroyltyramine	−9.91	54.14 nM
5281759	Caffeic acid 3-glucoside	−9.89	56.16 nM
5281787	Caffeic acid phenethyl ester	−9.34	143.37 nM
160355	Roscovitine	−9.16	193.30 nM
5919576	Benzyl caffeate	−8.8	354.66 nM
637540	o-Coumaric acid	−8.18	1.01 μM
5472440	Artepillin C	−7.59	2.72 μM
689043	Caffeic acid	−7.36	4.06 μM
689075	Methyl caffeate	−7.18	5.42 μM
819020	2-Methylcinnamic acid	−6.74	11.50 μM
445858	Ferulic acid	−6.69	12.38 μM
6440361	Drupanin	−6.26	25.80 μM
637542	p-Coumaric acid	−6.19	29.12 μM
444539	Cinnamic acid	−5.97	42.37 μM
637775	Sinapinic acid	−5.65	72.21 μM
446165	MBS (Ctrl+)	−9.30	152.20 nM
46505547	Marimastat (Ctrl+)	−8.65	454.89 nM

Abbreviations: Ctrl, control; *K*i, inhibition constant.

**Table 2:** Details of energies between top-ranked MMP3 inhibitors in this study, positive control inhibitors, and the enzyme's active site.

Ligand name	Final intermolcular energy (kcal/mol)	Final total internal energy (kcal/mol)	Torsional free energy (kcal/mol)	Unbound system's energy (kcal/mol)	Estimated free energy of binding (kcal/mol)
Cynarin	−9.16	−14.73	+6.26	−2.04	−15.57
Chlorogenic acid	−7.16	−11.03	+4.18	−1.52	−12.48
Rosmarinic acid	−8.45	−8.98	+4.47	−1.02	−11.93
Cinnamyl caffeate	−11.06	−1.88	+2.39	−0.51	−10.04
MBS (Ctrl+)	−11.42	−1.85	+2.98	−0.99	−9.3
Marimastat (Ctrl+)	−9.56	−3.47	+2.98	−1.39	−8.65

Abbreviation: MMP3, matrix metalloproteinase-3.

**Table 3:** Interactions between top-ranked 1HY7 inhibitors, positive control compounds, and MMP3 active site residues.

Ligand name	H-bond (Å)	Hydrophobic (Å)	Electrostatic (Å)
Cynarin (before MD)	Pro656 (4.28)	Val663 (6.14), His666 (5.10), His711 (7.55), Tyr668 (5.33)	NA

Cynarin (after MD)	Asp658 (3.58), Pro656 (4.57), Ala667 (3.72), His711 (4.36)	Phe586 (7.29), His711 (5.85), His666 (6.58)	NA

Chlorogenic acid	Val663 (3.15), Leu664 (5.11), Pro721 (5.40), Asn662 (6.28)	Val698 (4.81), His701 (4.07)	NA

Rosmarinic acid	Asn662 (5.35), His711 (4.76, 4.65, 5.39), Tyr723 (5.10)	His701 (5.03), Val698 (4.61), Val663 (4.61)	NA

Cinnamyl caffeate	His701 (4.53), Ala665 (3.31, 3.46), Leu664 (4.75)	His701 (4.72), Val698 (3.50), Leu697 (5.40, 4.70) Leu718 (5.72, 4.27), Leu726 (6.57), Ala665 (5.46), Tyr723 (4.40)	Glu702 (6.03)

MBS (Ctrl+)	His705 (5.84), His711 (5.64), Glu702 (5.96)	Leu697 (4.10, 6.14), Leu726 (5.69), Leu718 (5.40, 4.45), Val698 (4.44, 5.94), Val663 (4.82), His701 (4.20), Tyr723 (4.85)	NA

Marimastat (Ctrl+) (before MD)	Pro721 (4.87), Leu664 (3.47), Glu702 (5.05, 4.71)	Val698 (5.62), His701 (4.86), Leu718 (4.90), Val663 (4.98), Leu664 (4.57, 4.19), Ala665 (3.15), Leu722 (4.29)	NA

Marimastat (Ctrl+) (after MD)	His705 (4.82), Pro721 (4.48), Glu702 (4.78), Ala665 (3.3)	Leu697 (4.17), His701 (4.37, 5.43), Leu718 (4.03), Tyr720 (4.19)	Glu702 (7.38)

Abbreviations: MMP3, matrix metalloproteinase-3; NA, not available.

## Data Availability

The datasets used and/or analyzed during the current study are available from the corresponding author upon reasonable request.
